# Treatment of advanced cervical cancer with Cinobufacini and Paclitaxel–Cisplatin combination: A meta-analysis

**DOI:** 10.1097/MD.0000000000044146

**Published:** 2025-09-05

**Authors:** Zixin Chen, Lu Zhang, Die Li, Rui Zhu, Yue Luo

**Affiliations:** a Mianzhu City People’s Hospital, Mianzhu, Sichuan, China; b Mianzhu Chongqing University of Chinese Medicine, Chongqing, China.

**Keywords:** advanced cervical cancer, cinobufacini, paclitaxel-Cisplatin combination, meta-analysis

## Abstract

**Background:**

Standard treatments for advanced cervical cancer, such as paclitaxel–cisplatin combination (TP) chemotherapy, are often limited by reduced efficacy and significant toxicity. Cinobufacini (Huachansu), a traditional Chinese medicine, has demonstrated potential in enhancing the effectiveness of conventional cancer therapies.

**Methods:**

A systematic search of Web of Science, PubMed, Cochrane, Embase, China National Knowledge Infrastructure, and other databases was conducted up to July 30, 2024. Studies included were randomized controlled trials comparing cinobufacini combined with TP chemotherapy to TP chemotherapy alone in patients with advanced cervical cancer. The outcomes were clinical response rate, Karnofsky Performance Status, myelosuppression, platelet count, and incidences of vomiting and diarrhea. Data analysis was performed using RevMan 5.3, and risk ratios (RRs) and mean differences (MDs) were calculated with 95% confidence intervals (CIs). Heterogeneity was assessed using the *I*^2^ statistic, and sensitivity analysis was performed to ensure robustness.

**Results:**

Six randomized controlled trials involving 814 participants were included. Cinobufacini combined with TP chemotherapy significantly improved the clinical response rate (RR 1.22, 95% CI [1.05–1.41], *P* = .009) and KPS (MD 7.37, 95% CI [6.40–8.34], *P* < .00001). The intervention also reduced myelosuppression (RR 0.53, 95% CI [0.41–0.68], *P* < .0001), platelet count decline (MD −94.25, 95% CI [−96.96 to −91.52], *P* < .00001), vomiting (RR 0.58, 95% CI [0.45–0.76], *P* < .0001), and diarrhea (RR 0.60, 95% CI [0.39–0.92], *P* = .02). Heterogeneity was moderate for the clinical response rate but reduced after sensitivity analysis, with stable overall effect estimates.

**Conclusion:**

Cinobufacini combined with TP chemotherapy significantly improves clinical outcomes and reduces treatment-related adverse effects. These findings suggest that cinobufacini may be a valuable adjunctive therapy in enhancing the efficacy and reducing the toxicity of TP chemotherapy, though further large-scale studies are needed to confirm its efficacy and safety.

## 1. Introduction

The global incidence of gynecological malignancies among women has a marked disruptive effect on the functionality of the female reproductive system, leading to profound implications for their quality of life.^[1]^ Cervical, uterine, and ovarian cancers constitute the 3 primary subclasses within the gynecological cancer spectrum. Furthermore, statistics on mortality attributed to these malignancies reveal their significant impact.^[2,3]^ Cervical cancer is one of the most common gynecological malignancies, particularly in developing countries, where it remains a leading cause of cancer-related mortality.^[4–6]^ A significant proportion of patients are diagnosed at advanced stages, with the disease extending beyond the cervix.^[7–9]^ At this stage, cervical cancer often invades surrounding tissues and organs, causing symptoms such as lower abdominal distension, pelvic pain, and lumbosacral discomfort, which severely impact patients’ quality of life.^[10]^

The primary treatment modalities for advanced cervical cancer are radiotherapy and Paclitaxel and Cisplatin (TP) chemotherapy. However, these approaches face significant limitations.^[11–16]^ Advanced cervical cancer often demonstrates reduced sensitivity to many standard anticancer agents, resulting in suboptimal chemotherapy efficacy.^[17]^ Additionally, chemotherapy is associated with considerable toxicity, including myelosuppression and gastrointestinal side effects, which can hinder treatment adherence.^[18]^ Patients with advanced malignancies are also prone to hypercoagulable states, increasing the risk of thromboembolic events. These complications not only disrupt the treatment process but can further deteriorate patient outcomes and quality of life.^[19]^

In recent years, traditional Chinese medicine (TCM) has gained attention as an adjunctive therapy in the management of advanced cancers, including cervical cancer.^[20,21]^ TCM has demonstrated potential in alleviating symptoms, addressing hypercoagulability, and improving patients’ tolerance to conventional treatments such as radiotherapy and chemotherapy.^[22–36]^ Among TCM therapies, Cinobufacini (Huachansu) had emerged as a promising agent. Derived from the venom of the Bufo toad, Cinobufacini contains active compounds such as bufalin and cinobufagin, which exhibit potential anticancer properties.^[37–48]^ Preclinical and clinical studies suggest that Cinobufacini can boost the immune response, inhibit tumor growth, reducing chemotherapy-related toxicity, and improve survival rates in patients with various cancers, including liver and lung cancers.^[49,50]^ Its application in cervical cancer treatment offers a novel approach for enhancing patient outcomes when combined with standard therapies.^[42]^

Despite the widespread use of Cinobufacini in cancer treatment in China, its role in the management of advanced cervical cancer remains insufficiently studied.^[42,51–53]^ Preliminary research suggests that combining Cinobufacini with TP chemotherapy may offer synergistic effects, enhancing treatment outcomes while reducing chemotherapy-related toxicity. Given the limited reports on its use specifically for cervical cancer, this meta-analysis aims to systematically evaluate the efficacy of Cinobufacini in combination with TP chemotherapy for advanced cervical cancer. By analyzing relevant clinical studies, this work provides a comprehensive assessment of Cinobufacini’s potential benefits and limitations in this context.

## 2. Methods

### 2.1. Registration

This meta-analysis was conducted and reported according to the guidelines of Preferred Reporting Items for Systematic Reviews and Meta-Analyses. Research protocol was registered in the PROSPERO (No. CRD42024582993).^[54]^ And this study is a meta-analysis of previously published data. Ethical approval was waived by the Medical Ethics Committee of Mianzhu People’s Hospital, because it did not involve direct human subject participation.

### 2.2. Database and search strategy

The databases included Web of Science, PubMed, Cochrane, Embase, Wanfang database, China National Knowledge Infrastructure, and China Science and Technology Journal Database. Studies were searched from the inception of each database up to July 30, 2024, with language limited to Chinese or English. The search terms were MeSH combined with the keywords “Cinobufacini” or “Huachansu” or “Cinobupsin” and “cervical cancer.” Article references from relevant articles were reviewed to identify further pertinent publications.

### 2.3. Inclusion criteria include the following

Studies involving patients diagnosed with cervical cancer, specifically classified as stage IIB to IVA according to the International Federation of Gynecology and Obstetrics staging system, with no indications for surgery; studies where the experimental group received Cinobufacini combined with TP chemotherapy; studies where the control group received TP chemotherapy alone; studies that report at least 3 of the following outcome indicators: clinical response rate, Karnofsky Performance Status (KPS), incidence of myelosuppression, platelet count, incidence of vomiting, and incidence of diarrhea; studies that are randomized controlled trials (RCTs).

### 2.4. Exclusion criteria include the following

Studies involving patients with cervical cancer outside the Federation of Gynecology and Obstetrics IIB to IVA stages, or those with surgical indications; studies that do not use Cinobufacini combined with TP chemotherapy, or where different chemotherapy regimens are used; studies that do not include a control group receiving TP chemotherapy alone; studies that do not report at least three of the specified outcomes: clinical response rate, platelet count, KPS, incidence of vomiting, incidence of diarrhea, and incidence of myelosuppression; studies that are non-randomized, observational, case reports, reviews, editorials, or conference abstracts without full data.

### 2.5. Data acquisition

Two independent reviewers extracted data from the included studies using a pre-defined data extraction form. The data collected included: study characteristics including author, year of publication, country, and study design; patient characteristics including sample size, age, gender, and disease severity; intervention details encompassing types of treatments, dosage, and duration; and outcomes including clinical response rate, KPS, myelosuppression, platelet count, as well as incidence of vomiting, and diarrhea. Discrepancies between reviewers were resolved through discussion or by consulting a third reviewer.

### 2.6. Risk of bias assessment

Two reviewers (C.Z. and Z.L.) will independently assess risk of bias based on the following domains from recommendations from the Cochrane handbook: random sequence generation, allocation concealment, blinding of participants and persons, blinding of outcome assessment, incomplete outcome data, selective reporting, and other sources of bias. Each item was judged as low risk, high risk, and unclear. Each item was judged as low risk, high risk, and unclear. Results of bias assessment will be presented in figures. When 2 investigators disagreed, a consensus was reached through discussion. If no agreement could be reached, a third investigator (L.Y.) was asked to arbitrate.

### 2.7. Data analysis

Data analysis was performed using RevMan 5.3 (Cochrane, Oxford, United Kingdom). For continuous outcomes, such as KPS and platelet count, inverse variance method, and mean differences (MD) with 95% confidence intervals (CIs) were used for analysis. For dichotomous outcomes, including clinical response rate and the incidence of myelosuppression, vomiting, and diarrhea, Mantel–Haenszel method and risk ratios (RR) with 95% CIs were used. Statistical significance was determined with a *P*-value less than .05.

The heterogeneity among studies was assessed using the *I*² statistic, *I*² < 25% for no heterogeneity, 25% < *I*² < 50% for mild heterogeneity; 50% < *I*² < 75% is moderate heterogeneity, *I*² > 75% is severe heterogeneity. For outcomes with low or mild heterogeneity (*I*² < 50%), a fixed-effects model was applied. For outcomes with moderate or high heterogeneity (*I*² ≥ 50%), a random-effects model was used. This approach ensures that the statistical model chosen reflects the degree of variability observed across the studies. Heterogeneity was analyzed based on study design, intervention protocols, patient characteristics. Publication bias and potential outlier studies was evaluated using funnel plots. Sensitivity analyses were performed to assess the robustness of the results by excluding studies contributing disproportionately to the heterogeneity or with a high risk of bias.

## 3. Results

### 3.1. Literature search

**Figure 1.**
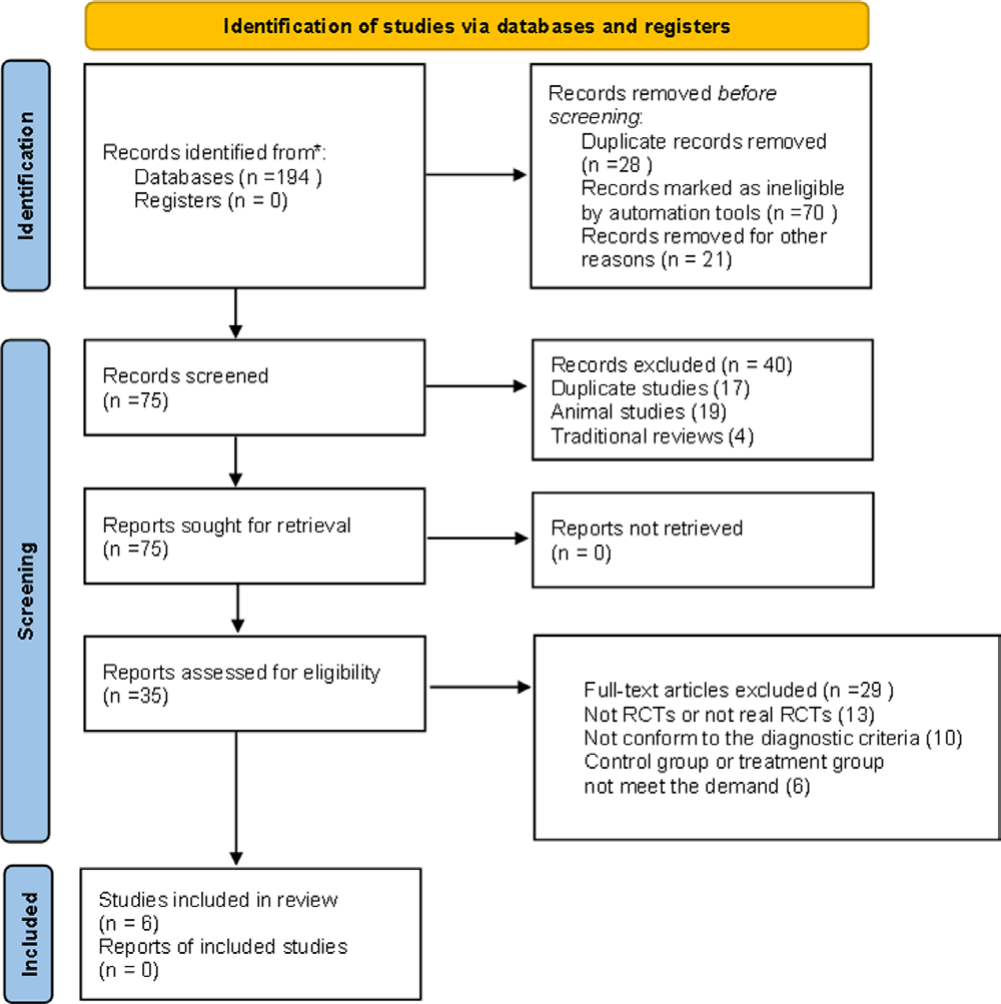
Flowchart of the search process and study selection. RCTs = randomized controlled trials.

As shown in Figure 1 and Table 1, a total of 194 articles were retrieved. Finally, there were 6 studies included with totaling 814 participants.^[55–60]^ And new studies were not identified via other methods. According to the Preferred Reporting Items for Systematic Reviews and Meta-Analyses, flow diagram produced with the web-based Shinyapp.

**Figure 2.**
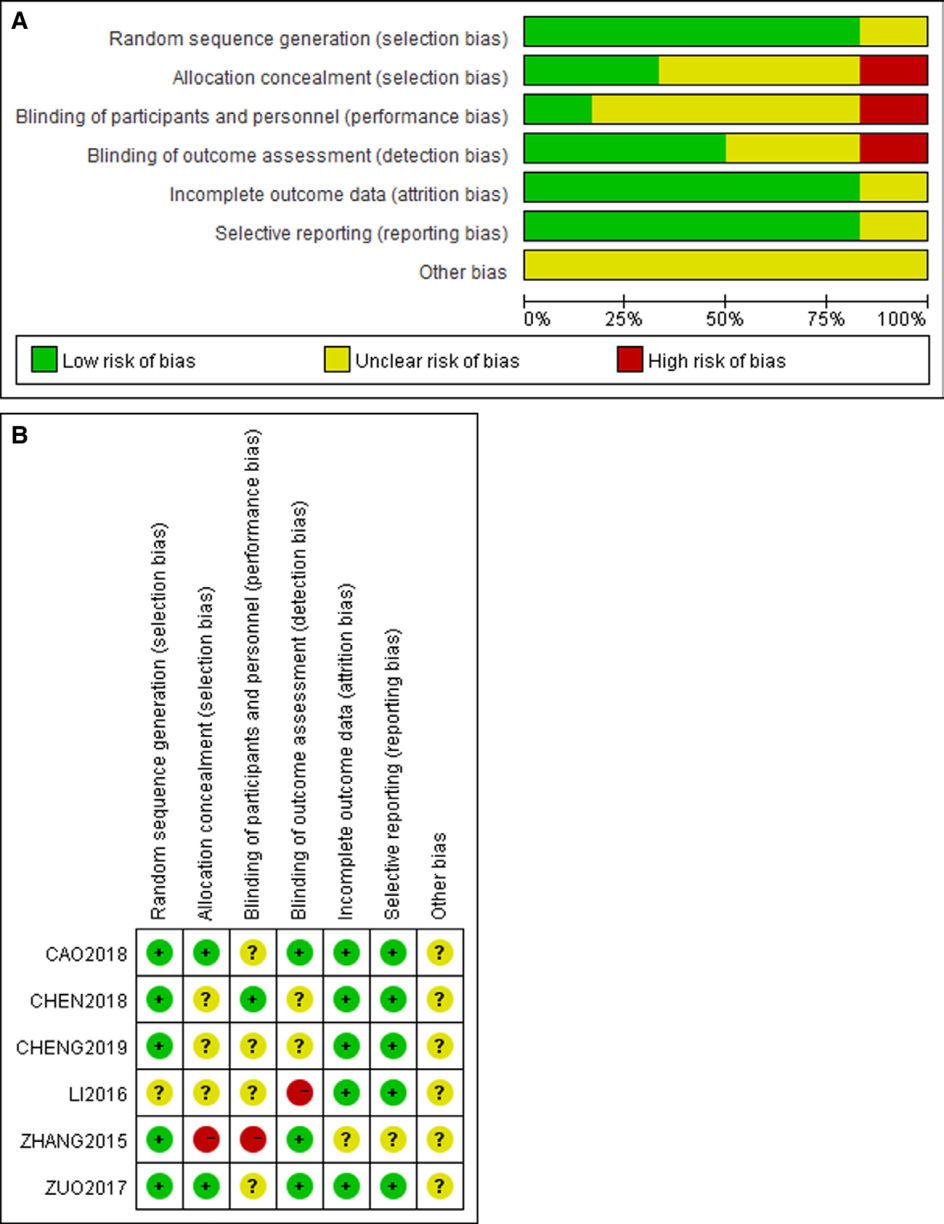
Overall risk of bias.

As showed in Figures 2, 5 studies^[56–58]^ reported random sequence generation, 1 study^[58]^ reported unclear. Allocation concealment methods were reported in 2 trials, but they were unclear in 3 trials,^[56–58]^ and 1 study^[59]^ had a high risk in this domain. Due to the difficulty of blinding participants and personnel to TCM interventions, only 1 study^[56]^ used blinding, 4 studies^[55,57,58,60]^ had unclear, and 1 study^[59]^ had a high risk in this domain. In terms of measurement bias, 3 studies^[55,59,60]^ used blinding to evaluate the study results, 2 studies^[56,57]^ had unclear blinding of outcome assessment bias, and 1 study^[58]^ had a high risk in this domain. Five studies^[55–58,60]^ were relatively complete and 1 study^[59]^ could be considered low risk in terms of incomplete outcome data. In terms of reporting bias, the research found that 5 studies^[55–58,60]^ were low risk, and 1 study^[59]^ had unclear. Furthermore, all studies were considered to have an unclear risk of other biases.

**Table 1:** Study characteristics for the included studies.

Study	Sample	Age range	Intervention		Course of treatment
	T/C	T/C	T	C	
Chen^[56]^	69/69	49.6 ± 5.3	Cinobufacini + TP	TP	6 W
Zhang^[59]^	30/30	48.3 ± 12.62	Cinobufacini	TP	3–6 W
Cheng^[57]^	36/36	58.9 ± 3.2	Cinobufacini + TP + Radiotherapy	TP + Radiotherapy	3–6 W
Zuo^[60]^	46/46	47.34 ± 2.57	Cinobufacini + TP + Radiotherapy	TP + Radiotherapy	6 W
Li^[58]^	60/57	35–75	Cinobufacini + TP	TP + Radiotherapy	8 W
Cao^[55]^	192/192	45.6 ± 8.4	Cinobufacini + TP	TP	4 W

### 3.2. Clinical response rate

**Figure 3.**
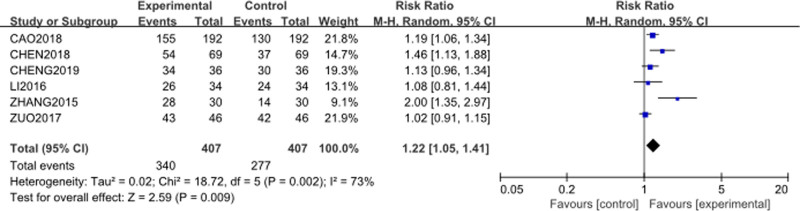
Forest plot of clinical response rate between Cinobufacini + TP versus TP alone. M–H = Mantel–Haenszel, TP = Paclitaxel–Cisplatin combination.

**Figure 4.**
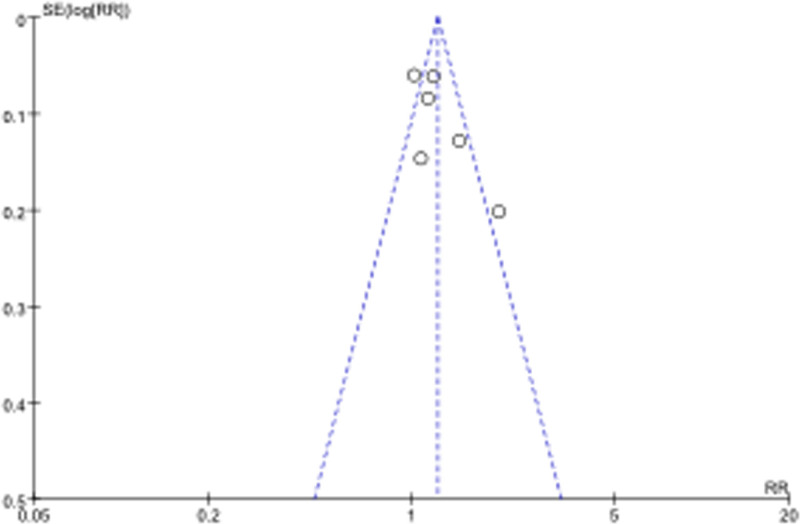
Funnel plot for publication bias assessment of clinical response rate.

The meta-analysis evaluated the clinical response rate between the intervention group (Cinobufacini + TP) and the control group (TP alone). Six studies^[55–60]^ were included, comprising 407 patients in the intervention group and 407 in the control group. The forest plot is presented in Figure 3, and the funnel plot is shown in Figure 4.

Heterogeneity among the studies was moderate (*P* = .002, *I*² = 73%), so the random-effects model was used. The RR for the clinical response rate was 1.22 (95% CI: [1.05–1.41], *P* = .009), indicating a statistically significant improvement in response with the addition of Cinobufacini.

**Figure 5.**
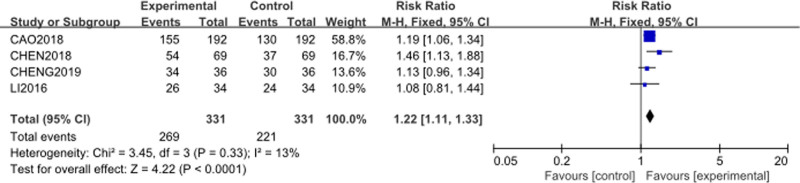
Forest plot of clinical response rate after excluding outlier studies. M–H = Mantel–Haenszel.

A sensitivity analysis was conducted by excluding 2 potential outlier studies. Funnel plot showed a slight degreeof asymmetry. Two points fell outside the 95% CI, which suggests that studies by Zhang^[59]^ and Zuo^[60]^ might be at risk of bias and could be considered outliers. After exclusion, the analysis was re-run using a fixed-effects model. The forest plot is presented in Figure 5. Heterogeneity decreased significantly (*P* = .33, *I*² = 13%), suggesting that these studies were the primary contributors to the initial heterogeneity. The RR was 1.22 (95% CI: [1.11–1.33], *P* = .0001), which remained virtually unchanged, indicating that the findings were robust.

This analysis suggests that the addition of Cinobufacini to TP chemotherapy consistently improves the clinical response rate.

### 3.3. KPS score

In the analysis of KPS score as an outcome, 3 studies^[56,57,60]^ were included, comprising a total of 302 patients, with 151 patients in the experimental group (Cinobufacini + TP) and 151 patients in the control group (TP alone).

**Figure 6.**
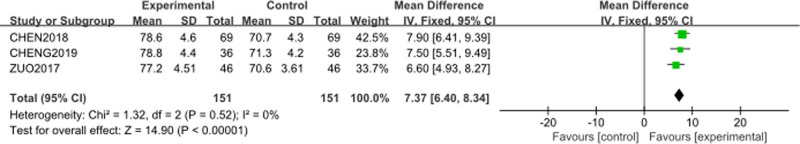
Forest plot of KPS improvement between Cinobufacini + TP versus TP alone. TP = Paclitaxel–Cisplatin combination.

The forest plot for KPS scores is presented in Figure 6. The results demonstrated a significant improvement in KPS scores in the Cinobufacini + TP group compared to the TP chemotherapy alone group. The MD was 7.37 (95% CI [6.40–8.34], *P* < .00001), indicating a highly statistically significant effect. Heterogeneity among the studies was very low (*P* = .52, *I*² = 0%), confirming high consistency across the studies. The result suggests that the addition of Cinobufacini to TP chemotherapy significantly enhances the KPS compared to TP chemotherapy alone.

### 3.4. Myelosuppression

**Figure 7.**
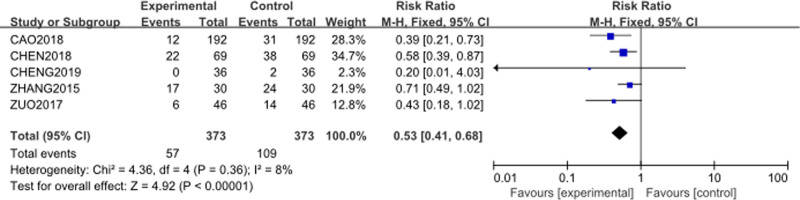
Forest plot of myelosuppression incidence between Cinobufacini + TP versus TP alone. M–H = Mantel–Haenszel, TP = Paclitaxel–Cisplatin combination.

The analysis included 5 studies^[55–57,59,60]^ with 373 patients in the intervention group (Cinobufacini + TP) and 373 in the control group (TP alone). The forest plot is presented in Figure 7. The RR for myelosuppression was 0.53 (95% CI [0.41–0.68], *P* < .0001), indicating a statistically significant difference. Heterogeneity among the studies was minimal (*P* = .36, *I*² = 8%), confirming high consistency across the studies. The result indicates that the addition of Cinobufacini to TP chemotherapy significantly reduces the risk of myelosuppression compared to TP chemotherapy alone.

### 3.5. Platelet count

**Figure 8.**
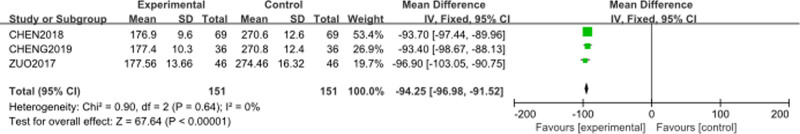
Forest plot of platelet count reduction between Cinobufacini + TP versus TP alone. TP = Paclitaxel–Cisplatin combination.

For the outcome of platelet count, 3 studies^[56,57,60]^ were included with 151 patients in the experimental group (Cinobufacini + TP) and 151 in the control group (TP alone). The forest plot is presented in Figure 8. The analysis demonstrated a significant reduction in platelet count decline in the experimental group compared to the control group, with a MD of −94.25 [95% CI, −96.96 to −91.52], *P* < .00001. Heterogeneity among the studies was very low (*P* = .64, *I*² = 0%), confirming high consistency across the studies. The result indicates that the addition of Cinobufacini to TP chemotherapy significantly reduces platelet count decline.

### 3.6. Vomiting

**Figure 9.**
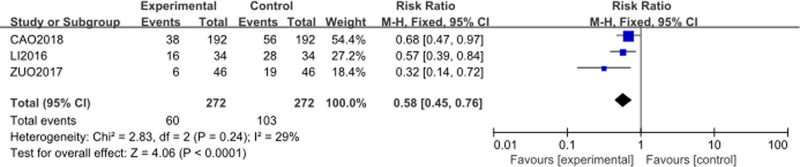
Forest plot of vomiting incidence between Cinobufacini + TP versus TP alone. M–H = Mantel–Haenszel, TP = Paclitaxel–Cisplatin combination.

The analysis included 3 studies^[55,58,60]^ with 272 patients in the intervention group (Cinobufacini + TP) and 272 patients in the control group (TP alone). The forest plot is presented in Figure 9. The RR for vomiting was 0.58 (95% CI [0.45–0.76], *P* < .0001) indicating a statistically significant difference. The heterogeneity among the studies was very mild (*P* = .24, *I*² = 29%). This suggests that the results are fairly consistent across studies. The analysis indicates that the addition of Cinobufacini to TP chemotherapy effectively mitigates the incidence of vomiting compared to TP chemotherapy alone.

### 3.7. Diarrhea

**Figure 10.**
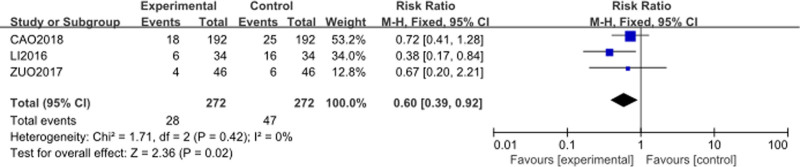
Forest plot of diarrhea incidence between Cinobufacini + TP versus TP alone. M–H = Mantel–Haenszel, TP = Paclitaxel–Cisplatin combination.

The analysis included 3 studies^[55,58,60]^ with 272 patients in the intervention group (Cinobufacini + TP) and 272 patients in the control group (TP alone). The forest plot is presented in Figure 10. The RR for diarrhea was 0.60 (95% CI [0.39–0.92], *P* = .02), indicating a statistically significant reduction in the risk of diarrhea in the intervention group compared to the control group. The heterogeneity was low, with a *P*-value of .42 and *I*² = 0%, suggesting consistency across the included studies. The result suggests that the addition of Cinobufacini to TP chemotherapy effectively mitigates the incidence of diarrhea compared to TP chemotherapy alone.

### 3.8. Publication bias analysis

Publication bias was assessed using a funnel plot for the clinical response rate outcome as shown in Figure 4. The funnel plot, which includes 6 studies,^[55–60]^ showed a slight degree of asymmetry. Two points were found outside the 95% CI, which suggests that studies by Zhang^[59]^ and Zuo^[60]^ might be at risk of bias and could be considered outliers. While the remaining 4 points were more symmetrically distributed around the overall estimate line, suggesting a relatively balanced representation of the included studies. A sensitivity analysis was conducted by excluding the 2 studies. The results are shown in Figure 5, which suggests that the overall effect estimate was robust to the removal of these studies. This bias analysis indicates that while publication bias may be a concern, the primary findings regarding the clinical response rate are consistent and reliable.

## 4. Discussion

This meta-analysis included 6 RCTs encompassing a total of 814 patients, providing robust evidence supporting the efficacy of Cinobufacini combined with TP chemotherapy in the treatment of advanced cervical cancer. The findings demonstrate significant improvements in key clinical outcomes when Cinobufacini is added to the standard TP chemotherapy regimen.

### 4.1. Principal findings

The most notable finding of this study is the significant increase in clinical response rate with an RR of 1.22, indicating that patients receiving Cinobufacini combined with TP chemotherapy had a 22% higher likelihood of achieving a clinical response compared to those treated with TP chemotherapy alone. Additionally, the KPS improved markedly with a MD of 7.37, suggesting that this combination therapy not only improves the effectiveness of the treatment but also enhances the quality of life of the patients.

Cinobufacini also appeared to mitigate some of the common adverse effects induced by TP chemotherapy. The incidence of myelosuppression was significantly lower (RR = 0.53), and the decline in platelet count was notably reduced (MD = −94.25). Furthermore, gastrointestinal adverse effects were alleviated, with significantly lower risks of vomiting (RR = 0.58) and diarrhea (RR = 0.60) in the Cinobufacini group, indicating better overall tolerability of the treatment.

Several previous meta-analyses have investigated the efficacy of Cinobufacini in combination with chemotherapy for other malignancies, such as gastric cancer and non-small cell lung cancer.^[52,61,62]^ These studies reported that Cinobufacini, when used alongside chemotherapy, could improve short-term clinical outcomes (including response rate and KPS) while also alleviating chemotherapy-induced adverse effects, such as leukopenia and vomiting. Our findings in advanced cervical cancer are in line with these prior results, demonstrating significant improvements in response rate and KPS, as well as reductions in myelosuppression, vomiting, and diarrhea. Taken together, these findings suggest that Cinobufacini may exert a consistent adjunctive effect when combined with chemotherapy across different tumor types, supporting its broader clinical potential. However, further high-quality, multicenter RCTs are still needed to confirm these results.

The results of the meta-analysis indicate that Cinobufacini, when used in combination with TP chemotherapy, can alleviate diarrhea and vomiting. However, some studies have reported that Cinobufacini monotherapy may cause mild diarrhea or low-grade cardiotoxicity in a small number of patients.^[63]^ The observed reduction in chemotherapy-induced diarrhea in our analysis may be attributed to a context-dependent effect: prior toxicities were primarily observed in high-dose monotherapy, whereas in the included studies, Cinobufacini was administered in low doses alongside chemotherapy. This suggests a possible synergistic and dose-modulating effect that warrants further investigation into its mechanism.

### 4.2. Heterogeneity and sensitivity analysis

In the analysis of the clinical response rate, heterogeneity was initially moderate (*I*² = 73%, *P* = .002), to investigate the source of this heterogeneity, a sensitivity analysis was conducted. Two potential outliers were suggested by the funnel plot analysis. After excluding the potential outlier studies by Zhang^[59]^ and Zuo,^[60]^ the heterogeneity was substantially reduced from 73% to 13% as shown in Figure 5, suggesting that these studies were the primary contributors to the initial heterogeneity. The observed heterogeneity may be attributed to differences in study design, intervention protocols, patient characteristics.

After the exclusion of the outlier studies, the pooled RR remained relatively unchanged at 1.22, indicating that the overall effect size is robust and is not unduly influenced by any single study or potential biases. For the other outcomes, including platelet count, KPS, myelosuppression, vomiting, and diarrhea, heterogeneity was very low. This consistency across outcomes reinforces the reliability of the observed effects. Therefore, the observed improvement in clinical response rate with the addition of Cinobufacini to TP chemotherapy is consistent and reliable.

### 4.3. Potential sources of heterogeneity

The sources of heterogeneity likely arise from variations in study design, patient characteristics, or differences in the implementation of treatment protocols across studies. The heterogeneity reduction after excluding the outlier studies indicates that specific methodological differences or variations in the application of Cinobufacini might have contributed to the observed inconsistencies. These findings highlight the importance of standardizing treatment protocols in future research to minimize heterogeneity.

### 4.4. Implications for practice

These results suggest that incorporating Cinobufacini into the TP chemotherapy regimen could be a valuable strategy for improving both clinical outcomes and the quality of life for patients with advanced cervical cancer. The reduction in adverse effects further supports its potential as a preferred adjunct therapy.

### 4.5. Limitations and future research

Several limitations should be acknowledged. First, the number of included studies and overall sample size remain limited, potentially affecting the statistical power and generalizability of the findings. And some included trials combined radiotherapy with chemotherapy. Although no substantial differences in treatment outcomes were observed between studies with and without radiotherapy, the small sample size and protocol heterogeneity prevented formal subgroup analyses. Therefore, the potential influence of radiotherapy on the observed efficacy of Cinobufacini cannot be fully excluded. In addition, not all included RCTs reported data for each outcome of interest, resulting in variability in the number of studies analyzed for each endpoint. This reflects the current lack of standardization in clinical outcome reporting for therapies involving Cinobufacini.

Second, all included studies were conducted in China, which may introduce regional bias. Genetic background, environmental factors, and healthcare practices specific to Chinese populations may influence treatment response. Future research should consider conducting multinational studies to explore potential population-specific effects and improve the external validity of the conclusions.

Third, although the results show a reduction in diarrhea and vomiting, the underlying mechanisms related to Cinobufacini’s dual role in both exerting mild toxicity and reducing chemotherapy-induced side effects remain unclear. Future studies should investigate the pharmacodynamic interactions between Cinobufacini and chemotherapy agents to better understand these effects.

Furthermore, a slight asymmetry in the funnel plot suggests the possibility of publication bias. Future studies should aim to include larger RCTs from diverse populations, with standardized treatment protocols to strengthen the evidence base and improve the applicability of findings.

## 5. Conclusion

In conclusion, Cinobufacini combined with TP chemotherapy shows significant promise in enhancing the clinical response rate and improving patient well-being by reducing the incidence of common chemotherapy-related adverse effects. The consistency of these findings across studies supports the potential for broader clinical adoption of this combination therapy in treating advanced cervical cancer.

## Acknowledgments

The authors would like to thank all individuals and institutions involved in this study. This research was supported by the Zhejiang Provincial Natural Science Foundation (Grant No. LQ21H270002), the Chongqing Natural Science Foundation Project (Grant No. CSTB2022NSCQ-MSX0652), and the Science and Technology Research Program of Chongqing Municipal Education Commission (Grant No. KJQN202215103).

## Author contributions

**Data curation:** Zixin Chen, Die Li.

**Investigation:** Zixin Chen, Lu Zhang, Die Li.

**Supervision:** Zixin Chen.

**Validation:** Rui Zhu.

**Visualization:** Zixin Chen, Rui Zhu.

**Writing – original draft:** Zixin Chen, Yue Luo.

**Writing – review & editing:** Lu Zhang, Die Li.
